# Exploring pain conceptualization in relation to outcomes in patients with chronic low back pain: a prospective observational study

**DOI:** 10.3325/cmj.2025.66.406

**Published:** 2025-12

**Authors:** Iva Lončarić Kelečić, Snježana Schuster

**Affiliations:** 1 University Hospital Center Zagreb, Zagreb, Croatia; 2 Department of Physiotherapy, University of Applied Health Sciences Zagreb, Zagreb, Croatia

## Abstract

**Aim:**

To examine the relationship between pain conceptualization and treatment outcomes in patients with chronic nonspecific low back pain (CNSLBP) undergoing physiotherapy, and to explore the influence of educational attainment on pain conceptualization.

**Methods:**

This prospective observational study enrolled 84 adults (mean age ≈50 years) receiving exercise-based physiotherapy for CNSLBP in an outpatient setting. Outcome measures included pain intensity (Numeric Pain Rating Scale), disability (Roland-Morris Disability Questionnaire), and health-related quality of life (EQ-5D-5L index and EQ-VAS), assessed before and after treatment. Pain conceptualization was measured with the Croatian version of the Concept of Pain Inventory for Adults (COPI-Adult).

**Results:**

Significant post-treatment improvements were observed in pain (−2.41), disability (−5.27), EQ-5D-5L index (+0.128), and EQ-VAS (+13.79) (all P < 0.001). Higher COPI-Adult scores were weakly and negatively correlated with pain (r = −0.273, P = 0.012) and disability (r = −0.259, P = 0.018), and positively with HRQoL (EQ-5D-5L: r = 0.295; EQ-VAS: r = 0.323). However, pain conceptualization did not significantly moderate the magnitude of outcome changes (all interaction effects P > 0.005). Participants with higher education scored significantly higher on COPI-Adult (P = 0.014; d = 0.55).

**Conclusions:**

While pain conceptualization is associated with post-treatment pain, disability, and HRQoL outcomes, it does not predict the degree of improvement after physiotherapy. Educational attainment influences pain conceptualization, which indicates a need for tailored communication strategies in rehabilitation. The Croatian COPI-Adult is a reliable tool for assessing patients' concepts of pain within CNSLBP care.

Chronic nonspecific low back pain (CNSLBP) is a common and burdensome condition affecting people of all ages and backgrounds worldwide. Musculoskeletal conditions are the most significant contributor to global rehabilitation needs, with low back pain (LBP) being the most prevalent (1). According to the Global Burden of Disease 2019 data, LBP accounts for 7.4% of global disability-adjusted life years (DALYs), ranking among the top ten causes of disability in many countries, including Croatia (2,3). Given its persistent nature and impact on work participation, CLBP leads to individual suffering and societal economic costs (4). The incidence and DALYs associated with LBP are expected to increase 1.4-fold by 2050 (5), which underscores the need for effective interventions.

CNSLBP is defined as pain in the lower back region lasting more than three months, without a specific identifiable cause (6,7). Unlike nociceptive pain with clear structural origins, CNSLBP is now considered a primary chronic pain disorder (8), stemming from a complex interplay of biological, psychosocial, and cultural factors (9,10). The experience of pain in these patients is not solely a physiological response but a subjective and dynamic process influenced by beliefs, emotions, and learned behaviors (11,12). In this context, a patient's conceptualization of pain, their understanding of its causes, meaning, and implications, emerges as a potentially modifiable determinant of health outcomes (13,14).

Clinical guidelines consistently endorse non-pharmacological, physiotherapy-led interventions as first-line treatment for CNSLBP, particularly structured exercises and patient education (15-17). A key educational component, pain neuroscience education (PNE), aims to reconceptualize and shift maladaptive pain beliefs, reduce fear-avoidant behaviors, and promote self-management (18-20). However, in Croatia, such educational approaches remain underutilized in clinical practice, despite evidence suggesting that pain misconceptions are prevalent among patients and healthcare professionals (21,22). Misunderstandings about pain origin, seriousness, and treatment may contribute to suboptimal outcomes and perpetuate disability (14).

While the relevance of pain reconceptualization in CNSLBP management has been increasingly recognized, limited research has examined the relationship between patients' pain conceptualization, as unprimed sense-making, and physiotherapy outcomes. Additionally, there is limited knowledge on how sociodemographic factors, such as educational attainment, influence these conceptualizations.

This study investigated the relationship between pain conceptualization and outcomes following physiotherapy in patients with CNSLBP. We hypothesized that (H1) pain conceptualization would be associated with pain outcomes, (H2) disability, and (H3) health-related quality of life (HRQoL). We also hypothesized that lower conceptualization would be related to (H4) less improvement in pain, (H5) disability, and (H6) HRQoL following physiotherapy. Additionally, we hypothesized that lower-educated patients would have lower pain conceptualization (H7).

## PARTICIPANTS AND METHODS

This pre-post observational prospective study enrolled CNSLB patients treated at the Department of Physiotherapy, University Clinical Hospital Center Zagreb, between January and June 2024. The study aligns with the principles established by Croatian and European regulations (23), including the Declaration of Helsinki (24). Informed consent from all participants was implied.

Participants were recruited through daily clinical practice and sampled via self-referral and consecutive recruitment (25) among patients with CNSLBP of both sexes. Eligible participants were adults aged 18-64 years with CNSLBP that had persisted for at least 3 months. Inclusion criteria were moderate pain intensity (≥4 on the Numeric Pain Rating Scale) (26), disability score ≥5 on the Roland Morris Disability Questionnaire (27), and no concurrent treatments for LBP aside from prescribed exercise or non-opioid systemic analgesics. Participants had to demonstrate adequate cognitive function, the ability to follow verbal instructions, and independently complete questionnaires. All patients were required to present a prior clinical evaluation and diagnosis report from a physician specialist confirming CNSLBP. Exclusion criteria were prior PNE including healthcare/medical education; clinical signs of radiculopathy; multilevel spinal pain; motor deficits; recent spinal trauma; neurological, psychiatric, or systemic conditions; pregnancy; osteoporosis; recent physiotherapy (within 3 months); use of opioid analgesics or excessive systemic analgesics; and any cognitive, sensory, or behavioral impairment that could interfere with participation or self-reporting. Patients without medical documentation, incomplete treatment, or assessment protocols were excluded. Participants were instructed not to use analgesic or anti-inflammatory medications during the ten-day physiotherapy program, except for their usual, pre-established dose as part of continuous therapy and, in case of need, paracetamol. Any increase in analgesic dosage or the use of opioid analgesics was not permitted.

The minimum sample size was determined using G*Power software (University of Düsseldorf, Germany, version 3.1.9.5/14 January 2020), which required at least 84 participants for an effect size of 0.35, a 5% type I error, and 80% power. Since consecutive sampling was a non-probability technique, data collection was verified for each participant (28).

### Research instruments

Sociodemographic data included sex (female/male), age (in years), and education level, stratified into lower (elementary/secondary) and higher (college/university). Clinical data included height (in cm), weight (in kg), disease duration (in months, categorized), prior physiotherapy (number of sessions, categorized), and medication use (type and current intake), which were recorded at baseline and monitored throughout treatment. Body height and weight values were used to calculate the body mass index (BMI) using the Metric BMI calculator (29).

For the pain outcome measurement, a Numeric Pain Rating Scale (NPRS) was used; a unidimensional measure of pain intensity ranging from 0 (no pain) to 10 (worst pain imaginable), which categorizes pain as mild (scores 1-3), moderate (scores 4-6), and severe (>7) (26,30). The NPRS has good sensitivity and generates data that can be statistically analyzed (31), with a two-point change representing a clinically meaningful change that exceeds the bounds of measurement error in patients with LBP (32).

The 24-item Roland-Morris Disability Questionnaire (RMDQ) (27) was used as a measure of disability outcome. The score ranges from 0 to 24 points, with a higher sum indicating a higher level of disability (27,33). The intraclass correlation of RMDQ ranges from 0.42 to 0.91 (34), and a threshold value of 4 best distinguishes changes in disability (35). This research used a Croatian version validated on persons with LBP (36).

HRQoL was assessed with the EQ-5D-5L, which comprises five domains (mobility, self-care, usual activities, pain/discomfort, and anxiety/depression) rated on a five-level scale, ranging from no problems to extreme problems, and a visual analogue scale (EQ-VAS) measuring the current health state (0-100) (37,38). Responses were converted from a five-digit code into an index value ranging from less than 0 (worse than dead) to 1 (full health), with higher scores indicating better health (39,40). Both the EQ-5D-5L index and EQ-VAS were used as outcome measures. Permission for the Croatian version was obtained (EuroQol ID: 60074), and the index was calculated using values from a similar neighboring country (41).

Pain conceptualization was assessed using the Croatian version of the Concept of Pain Inventory for Adults (COPI-Adult), adapted from Pate et al (42). The inventory comprises 13 items that explore individual beliefs about pain and its causes, which makes it suitable for individuals without prior PNE. Responses are rated on a 5-point scale (0 = strongly disagree to 4 = strongly agree), with total scores ranging from 0 to 52; higher scores indicate greater alignment with contemporary pain science. The original COPI-Adult demonstrated acceptable internal consistency (Cronbach’s alpha 0.78), with total score reliability estimated at 0.84 (42). In the Croatian sample, the scale showed good internal consistency (Cronbach’s alpha 0.803) (43). Instrument stability was assessed by comparing pre- and post-treatment scores.

### Physiotherapy treatment protocol

The quasi-experimental treatment protocol consisted solely of an exercise program for all participants. This included exercises for spine mobility, strengthening, and stabilization performed in sitting, prone, and supine positions. Exercises targeted the lumbar and pelvic muscles, incorporating proper breathing techniques, and were conducted daily for 10 consecutive days (excluding weekends), with each session lasting approximately 30 minutes. Participants performed exercises in supervised, individualized groups, as group exercise can increase motivation and reduce costs (44). Additionally, it is more effective in the short and long term, as indicated by patient-reported outcomes (45). Overall, the effectiveness of strength and stabilization exercises compared with other interventions in treating CLBP was confirmed by the best evidence (46,47). The absence of PNE was not a deliberate choice, as PNE is not included in standard approaches to treating CLBP in Croatia.

### Statistical analysis

The normality of data distributions was assessed using skewness and kurtosis statistics, with z-scores calculated by dividing each value by its standard error (48). For medium-sized samples (50 < n < 300), a threshold of |z| > 3.29 was used to reject the assumption of normality (49). Accordingly, significant skewness was observed in EQ-5D-5L index post (−7.274) and EQ-VAS post (−3.426), and significant kurtosis in EQ-5D-5L index post (10.992). Ceiling effects were evident in the EQ-5D-5L index post (value = 1) and the EQ-VAS post (value = 100), likely contributing to skewness (50). Given that EQ-5D-5L captures deviations from full health, such asymmetry was expected (51). Elevated kurtosis has also been reported in previous studies (52); despite these limitations, EQ-5D-5L remains acceptably responsive (51).

Descriptive statistics (mean, standard deviation, minimum, and maximum) were calculated for continuous variables, while categorical variables were summarized using frequencies and percentages. Outcome variables were treated as continuous, and COPI-Adult scores were analyzed both as continuous and dichotomized variables, as needed for statistical analysis. Because validated clinical cut-offs for the COPI-Adult are not available, and because categorization was required to allow two-factor modelling of group-by-time effects and interaction testing, COPI-Adult scores were dichotomized using a median split (lower vs higher conceptualization). This approach enabled the examination of whether baseline conceptualization moderated treatment-related changes.

Paired-samples t-tests were applied to compare pre- and post-treatment scores (53). Correlations between pain conceptualization and both post-treatment outcomes (H1, H2, and H3) and change scores (Δ) (54,55) (H4, H5, and H6) were examined using Pearson correlation coefficients (56), with the coefficient of determination (r^2^) reported to express explained variance (57). A correlation coefficient <0.20 indicated very weak correlation, 0.20-0.39 weak, 0.40-0.59 moderate, 0.60-0.79 strong, and >0.80 very strong. Additionally, univariate and multivariate analyses of variance were used to test the hypotheses H4, H5, and H6, examining both individual and combined effects of pain conceptualization on changes in pain, disability, and HRQoL outcomes. To assess the interaction between two pain conceptualization groups (0 = lower vs 1 = higher COPI-Adult) and time (pre-treatment vs post-treatment), a mixed ANOVA and a repeated-measures MANOVA were conducted, consistent with previously described procedures (58). Interaction effects and effect sizes were evaluated using partial eta squared (η^2^ₚ) (59,60). Examining the means, standard deviations, and sample sizes before ANOVA ensured that the statistical assumptions were met and results were valid (58). Between-group comparisons based on educational level (lower vs higher) (H7) were performed using independent-samples t-tests (53). A significance level of *P* < 0.005 was adopted for all tests, with additional attention given to findings at *P* < 0.001. Confidence intervals were set at 95% (61). The statistical analysis was conducted with SPSS Statistics for Windows, version 23.0 (IBM Corp., Armonk, NY, USA).

### Addressing potential sources of bias

The principal investigator conducted the protocol (screening, treatment, data collection, and processing). Pain conceptualization was controlled to ensure more accurate, potentially causal inferences (62). Clear inclusion criteria were applied to minimize confounding factors, and medical records were reviewed, with physician consultations as needed. Analgesic use was monitored daily. Self-referral and consecutive sampling were used to minimize bias (63). Analyses were conducted following the STROBE guidelines (64).

## RESULTS

Initially, 85 participants were assessed and deemed eligible for inclusion and underwent the treatment protocol. However, one participant missed the post-treatment assessment, so the final analysis included 84 participants. No further exclusions or dropouts occurred.

Patients’ mean age was approximately 50 years (range 22 to 64 years). Most participants were female (76.2%), and nearly an equal number of respondents had lower (48.8%) and higher education levels (51.2%) (Table 1). On average, participants had a BMI of 27.76. More than 65% experienced LBP for longer than three years. The majority (67.9%) had previously undergone LBP treatment, and among those who used over-the-counter pain medications, almost all (96.1%) relied on non-steroidal anti-inflammatory drugs. During treatment, two paracetamol doses were reported: 1000 mg for headache and 500 mg for menstrual pain.

**Table 1:** Basic sociodemographic and clinical characteristics of the sample*

Continuous variables	N	Min	Max	Mean	Standard deviation
**Age**	84	22	64	49.86	10.39
**Height**	84	150	192	169.80	8.9
**Weight**	84	46	130	80.76	19.58
**Body mass index**	84	17.6	44.6	27.76	7.78
**Categorical variables**	No. (of total 84)			%	
**Recruirement**					
self-referred	32			38	
convenient	52			62	
**Sex**					
male	20			23.8	
female	64			76.2	
**Level of education**					
Lower (elementary and secondary)	41			48.8	
Higher (college and university)	43			51.2	
**Pain duration**					
>3 to 6 months	10			11.9	
>6 to 12 months	5			5.9	
>1 to 3 years	14			16.7	
>3 to 10 years	30			35.7	
>10 years	25			29.8	
**Previous physiotherapy treatment for low back pain**					
yes	57			67.9	
no	27			32.1	
**Over-the-counter pain relief drugs**					
non-steroidal anti-inflammatory drugs	25			96.1	
analgetics/antipiretics	1			3.8	

*Characteristics may not sum up to 100% because of the effects of rounding.

After treatment, participants demonstrated significant improvements across all measured outcomes (Table 2). Pain intensity (NPRS) decreased by an average of 2.41 points (95% CI 2.08-2.74; *P* < 0.001). Disability (RMDQ) also improved significantly, with a mean decrease of 5.27 points (95% CI 4.51-6.03; *P* < 0.001). Regarding HRQoL, the EQ-5D-5L index scores increased by 0.128 points (95% CI 0.102-0.155; *P* < 0.001) and self-rated health (EQ-VAS) improved by 13.79 points (95% CI 11.70-15.89; *P* < 0.001).

**Table 2:** Differences in outcome measures pre- and post-treatment*

Pair	Paired differences					t-value	df	*P* (2-tailed)
	Mean	SD	SEM	95% CI				
				lower	upper			
**NPRS pre -NPRS post**	2.41	1.506	0.164	2.08	2.74	14.701	83	< 0.001
**RMDQ pre - RMDQ post**	5.27	3.503	0.382	4.51	6.03	13.797	83	< 0.001
**EQ-5D-5L index pre - EQ-5D-5L index post**	−0.128	0.123	0.013	−0.155	−0.102	−9.602	83	< 0.001
**EQ-VAS pre - EQ-VAS post**	−13.79	9.662	1.054	−15.89	−11.70	−13.087	83	< 0.001

*Abbreviations: NPRS – Numeric Pain Rating Scale; RMDQ – Roland Morris Disability Questionnaire; SD – standard deviation; SEM – standard error of the mean; DF – degrees of freedom; CI – confidence interval.

Pain conceptualization was significantly associated with all post-treatment outcomes (Table 3). Higher COPI-Adult scores were weakly and negatively correlated with pain intensity (r = −0.273, *P* = 0.012) and disability (r = −0.259, *P* = 0.018), and positively correlated with HRQoL, as measured by the EQ-5D-5L index (r = 0.295, *P* = 0.007) and EQ-VAS (r = 0.323, *P* = 0.003), confirming hypotheses H1, H2, and H3. COPI-Adult scores accounted for 7.5% of the variance in pain, 6.7% in disability, and 8.7% and 10.4% in HRQoL outcomes (index and VAS, respectively), providing theoretical support for the model (57).

**Table 3:** Correlations between pain conceptualization and pain, disability, and HRQoL outcome measures post-treatment*

		NPRS	RMDQ	EQ-5D-5L index	EQ-VAS
**COPI**	Pearson (*r*)	−0.273^†^	−0.259^†^	0.295^‡^	0.323^‡^
	*P* (2-tailed)	0.012	0.018	0.007	0.003

*Abbreviations: NPRS – Numeric Pain Rating Scale; RMDQ – Roland Morris Disability Questionnaire; HRQOL – health-related quality of life; VAS – visual analogue scale; COPI – Concept of Pain Inventory-Adult.

†Correlation is significant at the 0.005 level.

‡Correlation is significant at the 0.001 level.

**Figure 1:**
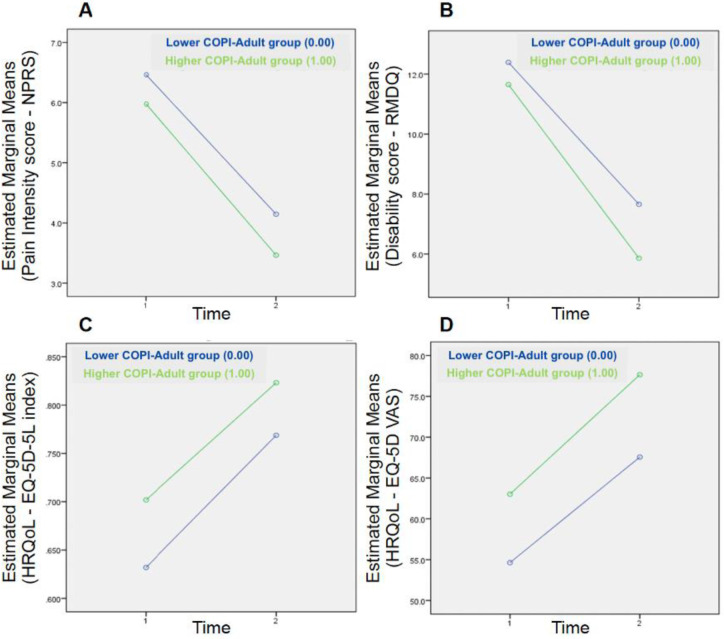
Profile plots of estimated marginal means pre- and post-treatment by COPI-Adult group for (A) pain intensity (NPRS), (B) disability (RMDQ), (C) HRQoL (EQ-5D-5L index), and (D) self-rated health (EQ-VAS). Lines represent estimated marginal means at two time points (pre- and post-treatment) for participants with lower and higher levels of pain conceptualisation (COPI-Adult group). Parallel lines indicate no significant interaction effect between time and the COPI-Adult group.

Results were more modest when examining the relationship of pain conceptualization with outcome changes. A small but statistically significant correlation was found between higher pain conceptualization and a greater reduction in disability (r = −0.232, *P* = 0.034). A similar negative correlation was observed for pain intensity (r = −0.212), although it was not significant (*P* = 0.053). No significant relationships were found between pain conceptualization and changes in EQ-5D-5L index (*P* = 0.231) or EQ-VAS (*P* = 0.396) (Table 4). Repeated-measures ANOVA performed on pain intensity (NPRS), disability (RMDQ), and HRQoL (EQ-5D-5L index and EQ-VAS), with dichotomized COPI-Adult scores (lower vs higher) as the between-subject factor (Table 5) revealed a significant main effect of time on pain intensity (F[1, 82] = 213.88, *P* < 0.001, partial η^2^ = 0.723), which indicates a substantial reduction following physiotherapy. However, the time × COPI interaction was non-significant (F[1, 82] = 0.35, *P* = 0.557, partial η^2^ = 0.004), which suggests similar improvements across both conceptualization groups. For disability, there was a significant main effect of time (F[1, 82] = 191.48, *P* < 0.001, partial η^2^ = 0.700), with a non-significant interaction of time and COPI (F[1, 82] = 1.94, *P* = 0.167, partial η^2^ = 0.023). Similarly, HRQoL improved significantly over time (EQ-5D-5L index (F[1, 82] = 91.66, *P* < 0.001, partial η^2^ = 0.528), but without significant interaction effects (EQ-5D-5L: F = 0.33, *P* = 0.567; EQ-VAS: F = 0.648, *P* = 0.423). Profile plots (Figure 1) confirm the absence of a significant time × COPI-Adult interaction, as indicated by the nearly parallel lines across all outcome measures.

**Table 4:** Correlations between pain conceptualization and delta differences for pain, disability, and HRQoL outcome measures

		NPRS Δ	RMDQ Δ	EQ-5D-5L index Δ	EQ-VAS Δ
**COPI**	Pearson (*r*)	−0.212	**-0.232** ^†^	−0.132	0.094
	*P* (2-tailed)	0.053	0.034	0.231	0.396

*Abbreviations: HRQOL – health-related quality of life; COPI – Concept of Pain Inventory-Adult pre-treatment; NPRS – Numeric Pain Rating Scale; RMDQ – Roland Morris Disability Questionnaire.

^†^Correlation is significant at the 0.005 level (two-tailed).

**Table 5:** Summary of within-subject effects of time and pain conceptualization on pain, disability, and health-related quality of life*^†^

Dependent variable	Time effect F(1,82), *P*	Partial η^2^ₚ	Time × COPI Interaction F(1,82), *P*	Partial η^2^
**Pain Intensity (NPRS)**	213.877, < 0.001	0.723	0.347, 0.557	0.004
**Disability** **(RMDQ)**	191.479, < 0.001	0.700	1.939, 0.167	0.023
**HRQoL (EQ-5D-5L Index)**	91.661, < 0.001	0.528	0.331, 0.567	0.004
**HRQoL** **(EQ-VAS)**	169.949, < 0.001	0.675	0.648, 0.423	0.008

*Abbreviations: HRQOL – health-related quality of life; COPI – Concept of Pain Inventory-Adult pre-treatment, NPRS – Numeric Pain Rating Scale; RMDQ – Roland Morris Disability Questionnaire*;* η^2^ₚ = partial eta squared.

†Repeated-measures ANOVA with time (pre/post) as within-subject factor and COPI-Adult group (lower/higher) as between-subject factor. No significant time × group interaction.

Repeated-measures MANOVA results (Table 6) showed a highly significant main effect of time (Wilks’ Lambda = 0.211, F[4, 79] = 74.035, *P* < 0.001), with a large effect size (partial η^2^ = 0.789), indicating substantial overall improvement post-treatment. The main effect of pain conceptualization group (COPI-Adult: lower vs higher) was not significant (Wilks’ Lambda = 0.910, F[4, 79] = 1.958, *P* = 0.109, partial η^2^ = 0.090), which suggested no overall differences in outcome profiles between groups. The time × COPI interaction was also non-significant (Wilks’ Lambda = 0.936, F(4, 79) = 1.354, *P* = 0.257, partial η^2^ = 0.064), which indicated that pain conceptualization did not moderate changes in outcomes over time. Based on these multivariate and corresponding univariate analyses, hypotheses H4, H5, and H6 were rejected.

**Table 6:** Multivariate effects of time and pain conceptualization on pain, disability, and health-related quality of life*^†^

Effect	Wilks’ Lambda	F (df)	*P*-value	Partial η^2^
**Time**	0.211	F(4, 79) = 74.035	< 0.001	0.789
**COPI-Adult Group**	0.910	F(4, 79) = 1.958	0.109	0.090
**Time × COPI Group**	0.936	F(4, 79) = 1.354	0.257	0.064

*Abbreviations: HRQOL – health-related quality of life; COPI – Concept of Pain Inventory-Adult pre-treatment; NPRS – Numeric Pain Rating Scale; RMDQ – Roland Morris Disability Questionnaire.

†Multivariate tests based on Wilks’ Lambda. Dependent variables included in the model: pain intensity (NPRS), disability (RMDQ), HRQoL (EQ-5D-5L index, and EQ-VAS). The COPI-Adult group (lower vs higher conceptualization) was used as a between-subjects factor. Partial η^2^ indicates effect size.

COPI-Adult scores significantly differed between individuals with higher and those with lower education (t = −2.219, *P* = 0.014; d = 0.55), falling within the range of medium to large effect (65) (Table 7). Given that lower-educated patients demonstrated significantly lower pain conceptualization compared with those with higher education, H7 was confirmed.

**Table 7:** Differences in Concept of Pain Inventory-Adult scores between participants with lower and higher-education

Group	N	Mean	Standard deviation	Cohen's d	t-value	*P*-value
**Lower education**	41	30.37	6.244	0.55	−2.219	0.014
**Higher education**	43	33.93	6.703			

*Significant at the 0.005 and 0.001 level.

In addition to good internal consistency of the Croatian COPI-Adult instrument (43), the COPI-Adult variables across two measurements exhibited a high level of stability, with a correlation coefficient of r = 0.943 and a significance level of *P* < 0.001; thus, the strong and significant correlation supports the use of COPI-Adult as a stable metric measurement.

## DISCUSSION

In this study, patients with more accurate pain conceptualization demonstrated better overall outcomes in terms of pain intensity, disability, and HRQoL. However, contrary to our hypotheses, pain conceptualization was not related to the degree of improvement over time. This suggests that while pain conceptualization is associated with overall outcomes, it may not influence the extent to which patients improve through exercise-based physiotherapy alone. While no directly comparable studies exist, our findings align with prior research showing that patients’ pain conceptualization is related to outcomes through processes of sense-making and reconceptualization. Considering the notion of the knowledge half-life, wherein older knowledge is increasingly replaced by newer findings (66), and acknowledging the hierarchy of evidence in evidence-based practice (67), this discussion prioritizes the most recent and methodologically robust studies relevant to the research problem.

A meta-analysis by Wood and Hendrick (68) found that improved pain conceptualization through PNE was associated with short-term reductions in pain and disability in patients with CLBP, although HRQoL was not directly assessed. Similarly, Ma et al (69) reported that combining PNE with exercise significantly reduced pain and disability, despite no observed changes in HRQoL. Umbrella reviews reinforce this pattern: Bonatesta et al (70) showed that PNE combined with exercise reduced pain, disability, kinesiophobia, and catastrophizing. Comparable conclusions were drawn by other studies (71-73), all of which emphasized the role of improved pain conceptualization in managing chronic musculoskeletal pain. While direct HRQoL effects remain underexplored, its established inverse relationship with pain and disability (74) lends further support to our findings.

Nevertheless, a critical perspective is necessary. As highlighted by Martinez-Calderon et al (75), the mixed quality and inconsistent findings of studies on PNE limit the firmness of clinical recommendations. While our results support the relevance of pain conceptualization, its relationship with patient outcomes remains only partially understood. COPI-Adult scores showed limited predictive value, explaining just 6.7% to 10.4% of the variance in outcomes. These proportions should be interpreted in the context of the short, exercise-only program, which likely constrained the variability required to observe stronger moderation effects. Contrary to expectations, pain conceptualization did not influence the degree of change, which suggests that exercise therapy was effective regardless of patients’ pain conceptualization. These findings are consistent with those of Ram et al (76), the only identified systematic review with a meta-analysis that directly examines the link between changes in pain knowledge and treatment outcomes in chronic musculoskeletal pain. Using the Neurophysiology Pain Questionnaire (NPQ) to assess knowledge, the review found no significant association between changes in pain knowledge and changes in pain intensity, disability, HRQoL, catastrophizing, or kinesiophobia. The authors propose that clinical improvement may be driven by the process that follows knowledge change, rather than the change itself. Our results support this view, suggesting that the focus should shift from measuring conceptual change to understanding how it shapes behavior and recovery. This has significant implications for shared decision-making in the treatment of CNSLBP. Given the need for cost-effective and time-efficient interventions that reduce work disability, physiotherapy should be targeted, purposeful, and responsive to individual patient factors, such as expectations, the need for diagnostic clarity, and healthcare usage patterns (77,78). Incorporating psychosocial and neuroplasticity-related elements may yield better outcomes than simply adding PNE to standard care (17).

Several factors may explain the lack of more substantial evidence in our findings. Some psychosocial factors, such as vigilance, fear, anxiety, depression, distress, and behavioral responses to pain, although controlled for, may have been overlooked. These factors have long been identified as significant predictors of ongoing pain and disability (79). Patient expectations, treatment motivation, perceived credibility, and self-efficacy are also influential (80), as are contextual factors such as therapeutic alliance (76). Psychological factors rooted in the fear-avoidance model have been linked to both pain and disability (81), while higher disability and anxiety levels are associated with lower HRQoL (82). Central sensitization (CS) has not yet been thoroughly studied as a predictor of treatment response in CNSLBP. However, evidence shows it correlates with worse outcomes in chronic musculoskeletal pain (83,84). Therefore, interventions should not rely solely on short-term improvements, especially in patients with a CS phenotype, where exercise therapy yields modest gains (85,86). Additionally, all the mentioned cognitive-emotional factors play a known role in CS (83,86). This suggests that deeper neurocognitive mechanisms may underlie the variability in outcomes.

Beyond filling a research gap, our results reaffirm the symbolic and practical significance of education in understanding the experience of pain. Education is a well-established determinant of health behavior and outcomes (87), with lower levels associated with greater pain intensity and poorer health outcomes (88). Our findings confirm that individuals with lower educational attainment have less aligned pain conceptualizations, offering further insight into vulnerable patient populations. Concerning the COPI-Adult inventory, it provides an inclusive, person-centered measure of pain knowledge and beliefs, validated for populations without prior education in pain science (42,43). Its good internal consistency was previously demonstrated (43), while our sample showed high test-retest reliability. In contrast, the NPQ and similar instruments used in previous research focus on neurophysiological knowledge, potentially overlooking psychological and social dimensions (42,76,89), and often assume a level of biomedical literacy that patients may not possess (90,91).

Based on our findings and existing literature, some hypotheses placed too much emphasis on pain conceptualization while underestimating psychosocial and neuroplasticity-related factors (92). Future research should integrate established theories with new empirical data, ideally through translational studies that bridge biomedical, social, and policy fields to improve patient care (93). Despite its strengths, this study has limitations: the correlational, pre-post observational design restricts causal inference, the monocentric and female-dominant sample may limit generalizability, and although COPI-Adult provides a more person-centered assessment than other tools, its predictive value should be further validated in longitudinal and interventional studies. Additionally, although several potential moderators (eg, age, sex, BMI, pain duration, medication use) were partly controlled through strict inclusion criteria and standardized procedures, they were not included in multivariable adjustment models, which may have allowed residual confounding. Several other methodological constraints should be considered: limited follow-up, dichotomization of COPI scores, and ceiling effects in HRQoL measures. Further research should incorporate a control group, ideally including an education-based comparator such as PNE, to enable clearer attribution of changes in outcomes and to evaluate whether baseline pain conceptualization interacts differently with educational versus exercise-based approaches. Aligned with a multidisciplinary approach, the potential synergy between the conceptualization of pain and the methodology of kinesiological anthropology creates opportunities for further analysis of morphological, functional, and behavioral factors related to CLBP. This approach could elucidate the role of physical activity and perceptual-cognitive processes within the context of personalized rehabilitation.

In conclusion, while pain conceptualization is associated with overall levels of pain, disability, and HRQoL in patients with CNSLBP undergoing physiotherapy, it does not affect the degree of improvement in these outcomes. Pain conceptualization did not significantly influence the magnitude of change in pain, disability, or HRQoL following treatment. These findings support informed clinical decision-making but also underscore a gap in addressing educational outcomes and broader health impacts in physiotherapy interventions.
